# Thai patients who fulfilled NCCN criteria for breast/ovarian cancer genetic assessment demonstrated high prevalence of germline mutations in cancer susceptibility genes: implication to Asian population testing

**DOI:** 10.1007/s10549-021-06152-4

**Published:** 2021-03-01

**Authors:** Pongtawat Lertwilaiwittaya, Ekkapong Roothumnong, Panee Nakthong, Peerawat Dungort, Chutima Meesamarnpong, Warisara Tansa-Nga, Khontawan Pongsuktavorn, Supakit Wiboonthanasarn, Warunya Tititumjariya, Wanna Thongnoppakhun, Sirisak Chanprasert, Chanin Limwongse, Manop Pithukpakorn

**Affiliations:** 1 Division of Medical Genetics, Department of Medicine, Faculty of Medicine Siriraj Hospital, Mahidol University, Bangkok, Thailand; 2 Siriraj Genomics, Faculty of Medicine Siriraj Hospital, Mahidol University, Bangkok, Thailand; 3 Siriraj Center of Research Excellence in Precision Medicine, Faculty of Medicine Siriraj Hospital, Mahidol University, Bangkok, Thailand; 4 Division of Medical Genetics, Department of Medicine, University of Washington, Seattle, USA

**Keywords:** Breast cancer, Germline genetic testing, Next Generation Sequencing

## Abstract

**Background:**

Germline genetic mutation plays a significant role in breast cancer susceptibility. The strength of such predisposition varies among ethnic groups across the globe, and clinical data from Asian population to develop a strategic approach to who should undergo a genetic test are lacking.

**Methods:**

We performed a multigene test with next generation sequencing in Thai patients whose clinical history fulfilled NCCN criteria for breast/ovarian cancer genetic assessment, consists of 306 breast cancer patients, 62 ovarian cancer patients, 14 pancreatic cancer patients and 7 prostate cancer patients. Genetic test result and clinical history were then checked with each NCCN criteria to determined detection rate for each indication.

**Results:**

There were 83 pathogenic/likely pathogenic (P/LP) variants identified in 104 patients, 44 of these P/LP variants were novel. We reported a high rate of germline P/LP variants in breast cancer (24%), ovarian cancer (37%), pancreatic cancer (14%), and prostate cancer (29%). Germline P/LP variants in *BRCA1* and *BRCA2* accounted for 80% of P/LP variants found in breast cancer and 57% of P/LP variants found in ovarian cancer. The detection rate of patients who fulfilled NCCN 2019 guideline for genetic/familial high-risk assessment of breast and ovarian cancers was 22–40%.

**Conclusion:**

Overall, the data from this study strongly support the consideration of multigene panel test as a diagnostic tool for patients with inherited cancer susceptibility in Thailand and Asian population. Implementation of the NCCN guideline is applicable, some modification may be needed to be more suitable for Asian population.

## Introduction

Breast cancer is the second most common cancer and the second leading cause of cancer-related death in the US [1]. Genetic predisposition accounts for 10–30% of breast cancer cases, and its rate of finding germline pathogenic variants in *BRCA1* or *BRCA2* (*gBRCA*) was 3–5% [2, 3]. In recent review, prevalence of *BRCA1*/*2* status in breast cancer varied across the globe. Mutations in *gBRCA* were found in 3% of unselected breast cancer, while the prevalence could be above 20% in selected group [4]. Following the discovery of *BRCA1* and *BRCA2*, several breast cancer genes with various degree of penetrance were identified [1]. *BRCA1*, *BRCA2*, *CDH1*, *STK11* and *TP53* are generally considered high-penetrance genes for breast cancer and the moderate-penetrance genes included *ATM*, *BRIP1*, *CHEK2*, and *PALB2,* though the gene lists can be dynamic [5, 6]. It is comprehensible that testing more genes could identify more patients with heritable form of breast cancer and provide benefit on cancer screening or prevention for at-risk individuals. With higher throughput and cheaper cost of next-generation sequencing, multigene panel testing has been widely adopted for patients with breast cancer [7].

Though, specific guidelines for management of each causative gene are increasingly available, consensus on breast cancer germline testing strategy among medical community at large is lacking. Various approaches on test eligibility are ranging from a population-based screening campaign to an universal genetic testing to an individual-based program [8]. Multiple models to estimate the greater-than-10% pretest likelihood of having *gBRCA* mutations and different testing criteria for patients with breast cancer have been used based on population data and national healthcare policies [9]. Successful clinical implementation of germline testing also requires data from ethnically diverse population. Unfortunately, existing models and test criteria are mostly suitable for Western population, while data on other ancestries are very limited.

This study aims to investigate prevalence and diversity of mutations from multigene panel testing of Thai patients with breast cancer and other related cancers in the hereditary breast-ovarian cancer spectrum and compare the clinical phenotype of patients with detectable mutations to a widely accepted clinical guideline.

## Materials and methods

### Study population

The study protocols were approved by the Siriraj Hospital Institutional Review Board Protocol No.474/2562(EC1) and 418/2562(EC2). The study was conducted according to the Good Clinical Practice and the Declaration of Helsinki. All Thai patients who were diagnosed with primary breast, ovarian, pancreas, or prostate cancers and treated at Siriraj Hospital, whose blood were sent for germline cancer susceptibility gene testing between 2016 and 2020 were included. We also included patients who had a report of pathogenic variants or likely pathogenic (P/LP) variants in genes for breast cancer (Table 1) as a secondary finding. We excluded patients with known clinical or molecular diagnosis of genetic diseases (e.g. neurofibromatosis type 1), patients referred for testing of only specific mutations, or asymptomatic individuals with known affected family members. We recruited a total of 377 unrelated consecutive patients. Three hundred and six patients had breast cancer, of which 19 of them also had primary ovarian cancer. Forty-three patients had primary ovarian cancer without breast cancer. There were 14 patients with pancreatic cancer and 7 patients with prostate cancer. Their tumor histological statuses, age of onset, and family history were comprehensively reviewed with the 2019 National Comprehensive Cancer Network (NCCN) guideline for genetic/familial high-risk assessment of breast and ovarian cancers. Descriptive statistics was used to calculate the rate of P/LP variants or variants of undetermined significance (VUS) across different indications. There was an additional recruitment of 7 patients who underwent multigene panel testing for another medical reason and harbored P/LP variants in causative genes for breast cancer (either *ATM, BRCA1* or *BRCA2*) as a secondary finding.

**Table 1:** List of genes tested in comprehensive cancer panel

Phenotype	Genes
High-penetrance gene for breast cancer	*BRCA1, BRCA2, CDH1, STK11, TP53*
Moderate-penetrance gene for breast cancer	*ATM, BRIP1, CHEK2, NF1, PALB2*
Possible breast cancer gene	*BARD1, NBN, RAD50, XRCC2*
Moderate-risk ovarian cancer gene	*RAD51C, RAD51D, MLH1, MSH2, MSH6, PMS2, EPCAM*
Genes that are highly penetrance in other types of cancer	*APC, AXIN2, BMPR1A, CDK4, CDKN2A, FANCC, MSH3, MUTYH, NTHL1, POLD1, POLE, PTEN, RECQL, SMAD4, VHL*

The OMIM numbers for each gene are *BRCA1* (OMIM number 113705)*, BRCA2* (OMIM number 600185)*, CDH1* (OMIM number 192090)*, STK11* (OMIM number 602216)*, TP53* (OMIM number 191170)*, ATM* (OMIM number 607585)*, BRIP1* (OMIM number 605882)*, CHEK2* (OMIM number 604373)*, NF1* (OMIM number 613113)*, PALB2* (OMIM number 610355)*, BARD1* (OMIM number 601593)*, NBN* (OMIM number 602667)*, RAD50* (OMIM number 604040)*, XRCC2* (OMIM number 600375)*, RAD51C* (OMIM number 602774)*, RAD51D* (OMIM number 602954)*, MLH1* (OMIM number 120436)*, MSH2* (OMIM number 609309)*, MSH6* (OMIM number 600678)*, PMS2* (OMIM number 600259)*, EPCAM* (OMIM number 185535)*, APC* (OMIM number 611731)*, AXIN2* (OMIM number 604025)*, BMPR1A* (OMIM number 601299)*, CDK4* (OMIM number 123829)*, CDKN2A* (OMIM number 600160)*, FANCC* (OMIM number 613899)*, MSH3* (OMIM number 600887)*, MUTYH* (OMIM number 604933)*, NTHL1* (OMIM number 602656)*, POLD1* (OMIM number 174761)*, POLE* (OMIM number 174762)*, PTEN* (OMIM number 601728)*, RECQL* (OMIM number 600537)*, SMAD4* (OMIM number 600993)*, VHL* (OMIM number 608537)

### Multigene panel test for hereditary cancer

Genomic DNA is extracted from peripheral blood. The DNA is enriched for the complete coding regions and splice junctions of the genes on this panel using custom-made targeted enrichment library. The list of genes tested in our panel is demonstrated in Table 1. All single nucleotide variants and copy number variants identified by multigene panel were validated with Sanger sequencing and Multiplex Ligation-dependent Probe Amplification (MLPA), respectively. The variants were interpreted and classified per 2015 ACMG-AMP standards and guidelines for the interpretation of sequence variants [10]. All reportable variants of each patient including pathogenic/like pathogenic variants (P/LP) and variants of undetermined significance (VUS) were manually verified. The detection rate of NCCN guideline indication fulfilment was calculated by dividing the number of patients with P/LP variants identified in each specific indication by the total number of patients who fulfilled the specific indication.

## Results

There were 83 unique P/LP variants identified in 104 patients (28.1%). Seventy-Three of 306 patients (23.9%) with breast cancer had germline P/LP variants. Twenty-Three of 62 patients (37.1%) with ovarian cancers carried germline P/LP variants. Two of 14 patients (14.3%) with pancreatic cancer harbored germline P/LP variants. Two of 7 patients (28.6%) with prostate cancer were identified with germline P/LP variants. Forty-Four out of 83 P/LP variants (53%) identified in this study have not been reported elsewhere. Thirty-One out of 57 (54%) *BRCA1* and *BRCA2* P/LP variants had not been previously reported. Meanwhile, VUS were found in 124 patients (41%) with breast cancer. Eight of them also had identified P/LP variants. VUS were observed in 21 patients (34%) with ovarian cancer, and six of them had identified P/LP variants. As for 14 patients with pancreatic cancer, 6 patients (43%) had VUS without any co-occurring P/LP variants. Four of 7 prostate cancer patients (57%) had VUS without any P/LP variants identified. No copy number variation (deletion/duplication) in cancer susceptibility gene was identified in this study.

### Mutation spectrum

Among 73 breast cancer patients with detectable P/LP variants, *BRCA1* and *BRCA2* accounted for 58 patients (79.5%). In 23 ovarian cancer patients with detectable P/LP variants, *BRCA1* and *BRCA2* accounted for 13 (56.5%) patients. *BRCA2* accounted for all 2 patients (100%) in 14 pancreatic cancer patients. *BRCA1* and *BRCA2* accounted for 2 patients (100%) in 7 prostate cancer patients.

Multigene panel targeted sequencing also identified germline mutations in genes other than *BRCA1* and *BRCA2*. Besides *BRCA1* and *BRCA2*, *ATM* (5 patients) was the most commonly mutated gene in this study followed by *PALB2* and *RECQL* (3 patients each). Other mutated genes included *APC, BRIP1, CHEK2, MLH1, MSH2, PMS2, MUTYH, NBN, RAD51C, RAD51D*, and *TP53*.

From 219 VUS identified in this study, only 27 VUS (12.3%) were found in *BRCA1* and *BRCA2* while 192 VUS belonged to other genes. *ATM* was the most commonly identified gene with VUS, followed by *APC* and *MSH6*. There were 7 putative loss-of-function (pLOF) variants (frameshift, stop gain, start loss, and splice site variants) in 6 genes (*APC, BRCA2, MSH2, RECQL, RAD51C*, and *XRCC2*) with insufficient data to be designated as P/LP.

**Fig. 1:**
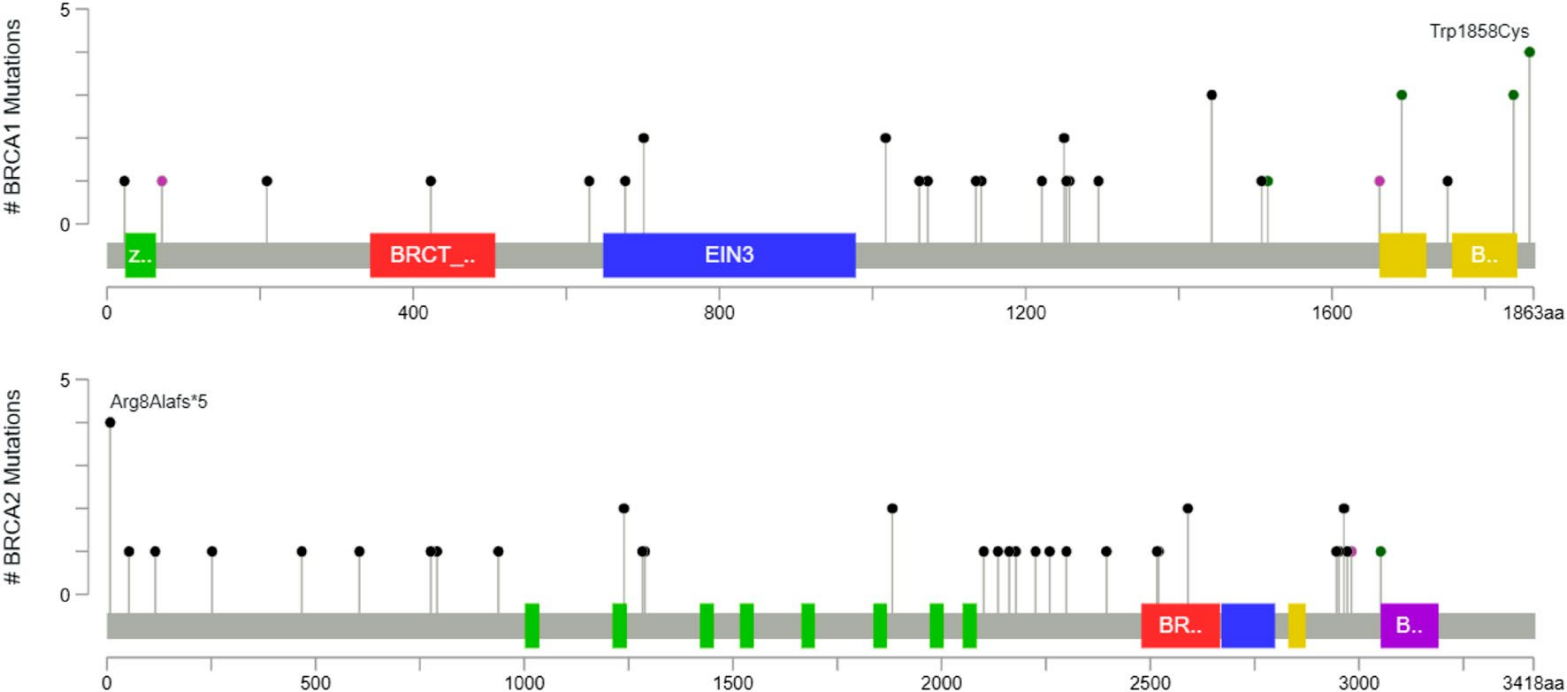
Lollipop plot of P/LP variants in *BRCA1* and *BRCA2*

The details of identified P/LP variants, patient’s phenotype and family history were shown in Table 2. We also included 7 patients who had a report of P/LP variants in genes for breast cancer as a secondary finding in Table 2. Details of VUS with putative loss-of-function prediction and its patient’s phenotype were listed in Table 3. All P/LP variants in *BRCA1*/*2* were illustrated in a lollipop plot in Fig. 1.

**Table 2:** List of gene(s), variants, classification, and patient’s history

Gene (Reference sequences)	Variant nomenclature	Variant classification	Cancers diagnosed in the patient	Cancers diagnosed in family member(s)
*APC *(NM_000038.5)	c.1620dupA, p.Gln541Thrfs*19	**Pathogenic**	**Ovary**	**–**
	c.2977_2980delAAGT, p.K993Ffs*11	**Likely Pathogenic**	**Breast**	**Breast**
*ATM *(NM_000051.3)	c.875C > T, p.Pro292Leu	Likely Pathogenic	Ovary	–
	c.2086G > T, p.Gly696*	**Likely Pathogenic**	**Eye**	–
	c.3693_3697delATCTT, p.Leu1231Phefs*13	**Pathogenic**	**Ovary**	–
	c.7519_7520delGA, p.Asp2507Argfs*8	**Likely Pathogenic**	**Colon**	–
	c.8434_8435delTC, p.Ser2812Phefs*2	Likely Pathogenic	Colon, Common bile duct	Stomach, Liver, Pancreas
*BRCA1 *(NM_007294.3)	c.68_69delAG, p.Glu23Valfs*17	Likely Pathogenic	Colon	
	**c.213-12A > G**	**Pathogenic**	**Breast**	**Breast**
	c.624_625ins(20), p.Pro209Argfs*32	**Pathogenic**	**Breast**	**Breast**
	c.1265_1266dupAT, p.Ser423Ilefs*8	**Pathogenic**	**Ovary**	**Ovary, Lung**
	c.1504_1508delTTAAA, p.Leu502Alafs*2	Pathogenic	Breast	Breast, Ovary
	c.1889delA, p.Asn630Ilefs*2	**Pathogenic**	**Breast**	**Breast**
	c.2101_2102delAA, p.Lys701Valfs*10	**Pathogenic**	**Breast**	**Breast**
	c.3049G > T, p.Glu1017*	**Pathogenic**	**Prostate**	**Stomach, Prostate, Pancreas, Thyroid**
	c.3049G > T, p.Glu1017*	**Pathogenic**	**Breast**	
	c.3181delA, p.Ile1061*	**Pathogenic**	**Ovary**	**Bladder**
	c.3214delC, p.Leu1072*	**Pathogenic**	**Breast**	**Pancreas, Ovary**
	c.3403C > T, p.Gln1135*	Pathogenic	Breast, Ovary, Thyroid	Ovary
	c.3424delG, p.Ala1142Hisfs*13	Pathogenic	Breast, Ovary	Breast
	c.3661G > T, p.Glu1221*	Pathogenic	Breast	Pancreas, Ovary, Unknown
	c.3748G > T, p.Glu1250*	Pathogenic	Breast	Breast, Ovary
	c.3748G > T, p.Glu1250*	Pathogenic	Breast	Breast
	c.3756_3759delGTCT, p.Ser1253Argfs*10	Pathogenic	Breast, Ovary	Breast
	c.3770_3771delAG, p.Glu1257Glyfs*9	Pathogenic	Ovary	Ovary
	c.3882_3885delCTTG, p.Leu1295Phefs*11	**Pathogenic**	**Breast, Ovary**	**Breast**
	c.4327C > T, p.Arg1443*	Pathogenic	Breast	Breast, Ovary
	c.4327C > T, p.Arg1443*	Pathogenic	Breast	–
	c.4327C > T, p.Arg1443*	Pathogenic	Breast	–
	c.4484G > A, p.Arg1516Lys	Likely Pathogenic	Breast	Breast, Colon
	c.4523G > A, p.Trp1508*	Pathogenic	Breast, Ovary	Peritoneum
	**c.4986 + 1G > T**	**Pathogenic**	**Ovary**	**Breast, Ovary**
	c.5030_5033delCTAA, p.T1677Ifs*2	**Pathogenic**	**Breast**	**Breast**
	**c.5072C > A, p.Thr1691Lys**	**Likely Pathogenic**	**Lung**	**Pancreas, Breast, Ovary**
	**c.5072C > A, p.Thr1691Lys**	**Pathogenic**	**Breast**	**Breast**
	**c.5072C > A, p.Thr1691Lys**	**Pathogenic**	**Ovary**	**Breast**
	c.5251C > T, p.Arg1751*	Pathogenic	Breast	–
	**c.5511G > T, p.Trp1837Cys**	**Likely Pathogenic**	**Breast**	–
	**c.5511G > T, p.Trp1837Cys**	**Likely Pathogenic**	**Breast**	–
	**c.5511G > T, p.Trp1837Cys**	**Likely Pathogenic**	**Breast**	–
	c.5574G > T, p.Trp1858Cys	Likely Pathogenic	Breast	Ovary
	c.5574G > T, p.Trp1858Cys	Likely Pathogenic	Breast	Breast
	c.5574G > T, p.Trp1858Cys	Likely Pathogenic	Breast	Breast
	c.5574G > T, p.Trp1858Cys	Likely Pathogenic	Breast	Breast
*BRCA2 *(NM_000059.3)	c.18_19delAG, p.Arg8Alafs*5	Pathogenic	Breast	–
	c.22_23delAG, p.Arg8Alafs*5	**Pathogenic**	**Breast**	–
	c.22_23delAG, p.Arg8Alafs*5	**Pathogenic**	**Breast, Ovary**	–
	c.22_23delAG, p.Arg8Alafs*5	**Pathogenic**	**Breast**	**Breast**
	c.22_23delAG, p.Arg8Alafs*5	**Pathogenic**	**Pancreas**	**Breast**
	c.157A > T, p.Lys53*	**Pathogenic**	**Breast**	**Breast**
	c.346delA, p.Ser116Valfs*5	**Pathogenic**	**Breast**	**Breast, Ovary, Prostate**
	c.755_758delACAG, p.Asp252Valfs*24	**Pathogenic**	**Nasopharynx**	**Breast, Ovarian, Pancreas**
	c.1399_1402delAAGA, p.Lys467Glufs*17	Pathogenic	Breast	Breast
	c.1813delA, p.Ile605Tyrfs*9	Pathogenic	Colon	Colon, Breast, HCC
	c.2327delA, p.Lys776Argfs*7	Pathogenic	Ovary	–
	c.2372C > G, p.Ser791*	**Pathogenic**	**Breast**	–
	c.2808_2811delACAA, p.Ala938Profs*21	**Pathogenic**	**Breast**	**Male breast, Ovary**
	c.3716_3717delAA, p.Lys1239Thrfs*3	Pathogenic	Breast	Breast, Leukemia, Prostate
	c.3716_3717delAA, p.Lys1239Thrfs*3	Pathogenic	Colon	Lung
	c.3847_3848delGT, p.Val1283Lysfs*2	Pathogenic	Breast, Ovary	Peritoneum
	c.3865_3868delAAAT, p.Lys1289Alafs*3	**Pathogenic**	**Breast**	**Breast**
	c.5645C > A, p.Ser1882*	Pathogenic	Breast, Thyroid	Prostate
	c.5645C > A, p.Ser1882*	Pathogenic	Breast	Breast
	c.6298_6299insA, p.Asn2101Lysfs*10	Pathogenic	Breast	Breast, Endometrium, Pancreas
	c.6405_6409delCTTAA, p.Asn2135Lysfs*3	**Pathogenic**	**Breast**	–
	C6486_6489delACAA, p.Lys2162Asnfs*5	Pathogenic	Breast	Breast
	c.6532dupC, p.His2178Profs*11	**Pathogenic**	**Breast**	**Breast, Prostate, Colon**
	c.6673delA, p.Thr2225Glnfs*4	**Pathogenic**	**Breast**	**Breast**
	c.6777_6778delTG, p.N2259Kfs*33	**Pathogenic**	**Breast, Ovary**	**Colon, Endometrium**
	c.6896delA, p.Asn2299Ilefs*6	**Pathogenic**	**Breast**	–
	c.7185_7188delCTTG, p.His2395Glnfs*71	**Pathogenic**	**Breast**	**Breast**
	c.7544_7545insA, p.Ser2516Ilefs*23	**Pathogenic**	**Breast**	**Unknown metastasis**
	c.7558C > T, p.Arg2520*	Pathogenic	Pancreas	–
	c.7767delC, p.Ser2590Profs*58	**Pathogenic**	**Breast, Endometrium**	**Breast, Thyroid**
	c.7767delC, p.Ser2590Profs*58	**Likely Pathogenic**	**Breast**	–
	c.8837_8841delTGGAA, p.Leu2946Tyrfs*2	**Pathogenic**	**Prostate, Male breast, Esophagus**	–
	c8854_8855insT, p.Met2952Ilefs*5	**Pathogenic**	**Breast**	**Breast, Colon, Ovary**
	c.8890dupA, p.Arg2964Lysfs*54	**Pathogenic**	**Breast**	–
	c.8890dupA, p.Arg2964Lysfs*54	**Pathogenic**	**Breast, Ovary**	–
	c.8915delT, p.Leu2972Cysfs*4	**Pathogenic**	**Breast**	**Breast**
	c.8953 + 1G > C	Likely Pathogenic	Breast, Ovary	Breast
	c.9154C > T, p.Arg3052Trp	Pathogenic	Breast	Breast, Ovary
*BRIP1 *(NM_032043.2)	c.1343G > A, p.Trp448*	Pathogenic	Breast	–
	c.2431_2432dupCT, p.Pro812Tyrfs*15	Likely Pathogenic	Breast	Breast
*CHEK2 *(NM_007194.3)	**c.1008 + 2 T > A**	**Likely Pathogenic**	**Breast**	–
*MLH1 *(NM_000249.3)	c.790 + 1G > A	Pathogenic	Ovary, Endometrium	Breast, Endometrium
*MSH2 *(NM_000251.2)	c.811_814delTCTG, p.Ser271Argfs*2	**Pathogenic**	**Breast**	–
	c.1237C > T, p.Gln413*	**Pathogenic**	**Ovary**	**Endometrium, Breast**
*MUTYH *(NM_001128425.1)	c.934-2A > G	Likely Pathogenic	Breast	–
*NBN *(NM_002485.4)	c.89delA, p.Asn30Thrfs*5	**Likely Pathogenic**	**Breast**	–
*PALB2 *(NM_024675.3)	c.2968G > T, p.Glu990*	Pathogenic	Breast	Breast
	c.3267_3268delGT, p.Phe1090Serfs*6	**Pathogenic**	**Ovary**	**Lung**
	c.3426_3429delAAGT, p.Leu1142Phefs*20	Pathogenic	Breast	Breast
*PMS2 *(NM_000535.6)	c.325dupG, p.Glu109Glyfs*30	Pathogenic	Ovary	–
	**706-1G > T**	**Pathogenic**	**Breast**	**Breast**
*RAD51C*	c.905-2A > C	Likely Pathogenic	Breast	Breast
*RAD51D *(NM_002878.3)	c.270_271dupTA, p.Lys91Ilefs*13	Pathogenic	Ovary	–
	c.270_271dupTA, p.Lys91Ilefs*13	Pathogenic	Breast	Male Breast
*RECQL *(NM_002907.3)	c.796C > T, p.Gln266*	Pathogenic	Breast, Ovary	Breast
	c.796C > T, p.Gln266*	Pathogenic	Breast	Ovary
	c.1217-2A > C	Likely Pathogenic	Breast, Ovary	–
*TP53 *(NM_000546.5)	**c.96 + 1 G > A**	**Pathogenic**	**Breast, Brain**	**Breast, Lung**
	c.1024C > T, p.Arg342*	**Pathogenic**	**Breast**	–

The variants highlighted in bold are novel variants (not previously observed in control or mutation databases or published articles)

**Table 3:** List of putative loss-of-function VUS (frameshift deletion, stop gain, start loss, splice site variant)

Gene (Reference sequences)	Variant nomenclature	Variant classification	Cancers diagnosed in the patient
*APC*	c.1A > G, p.Met1Val	Start loss	Ovary
*BRCA2*	c.8954-5_8954-2delAACA	Splice variant	Ovary
	c.7617 + 2dupT	Splice variant	Primary Peritoneal
*MSH2*	c.792 + 3A > T	Splice variant	Breast
*RECQL*	c.2 T > C, p.Met1Thr	Start loss	Breast
*RECQL*	c.2 T > C, p.Met1Thr	Start loss	Breast
*RAD51C*	c.571 + 5G > A	Splice variant	Breast
*XRCC2* (NM_005431.1)	c.832G > T, p.Glu278*	Stop gain	Breast

### Multigene panel testing in breast cancer categorized by NCCN 2019 indication fulfillment

**Fig. 2:**
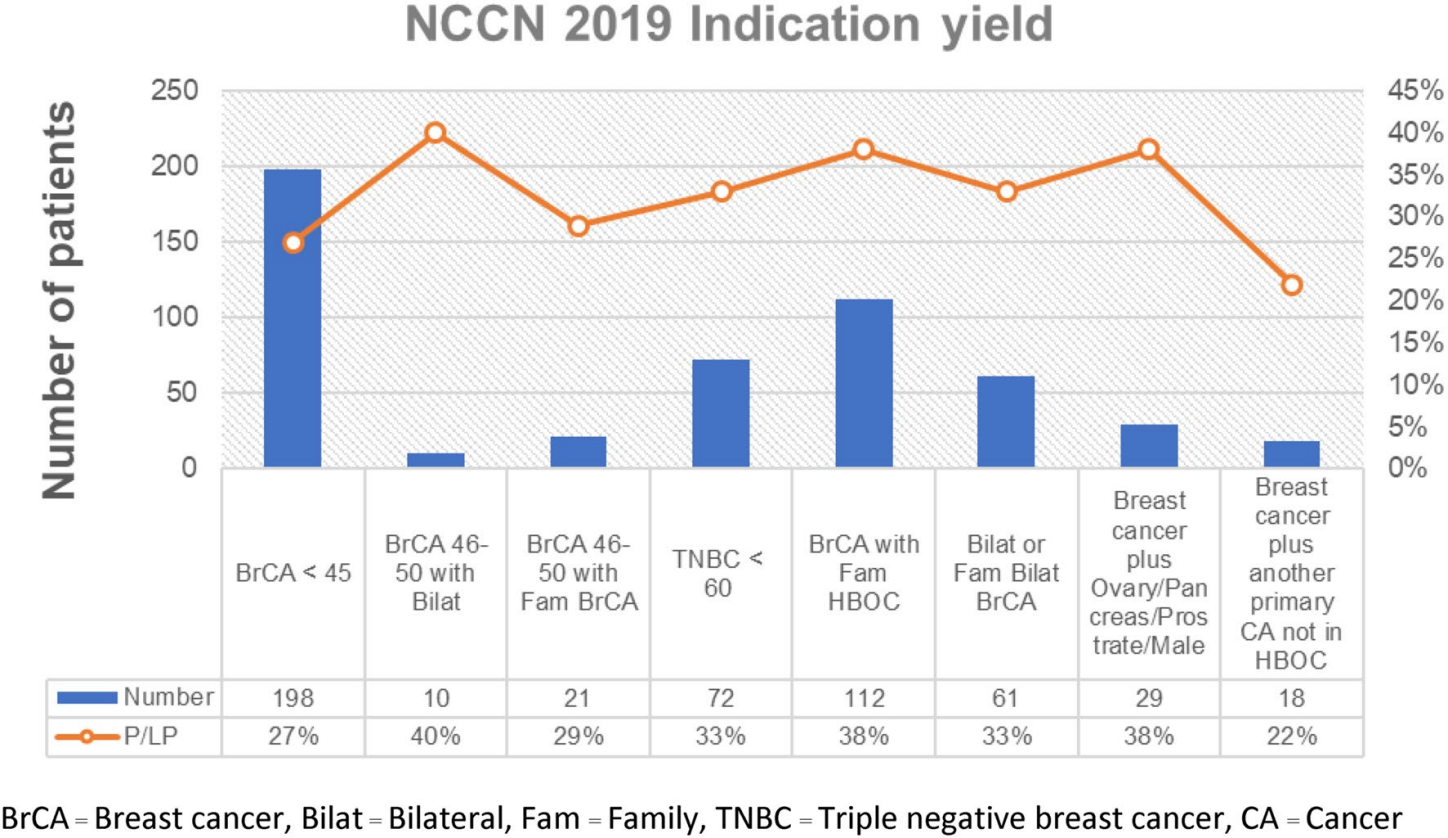
Rate of germline pathogenic/likely pathogenic variants or VUS from multigene panel test in Thai patients with breast cancer. *BrCA* Breast cancer, *Bilat* Bilateral, *Fam* Family, *TNBC* Triple negative breast cancer, *CA* Cancer

**Fig. 3:**
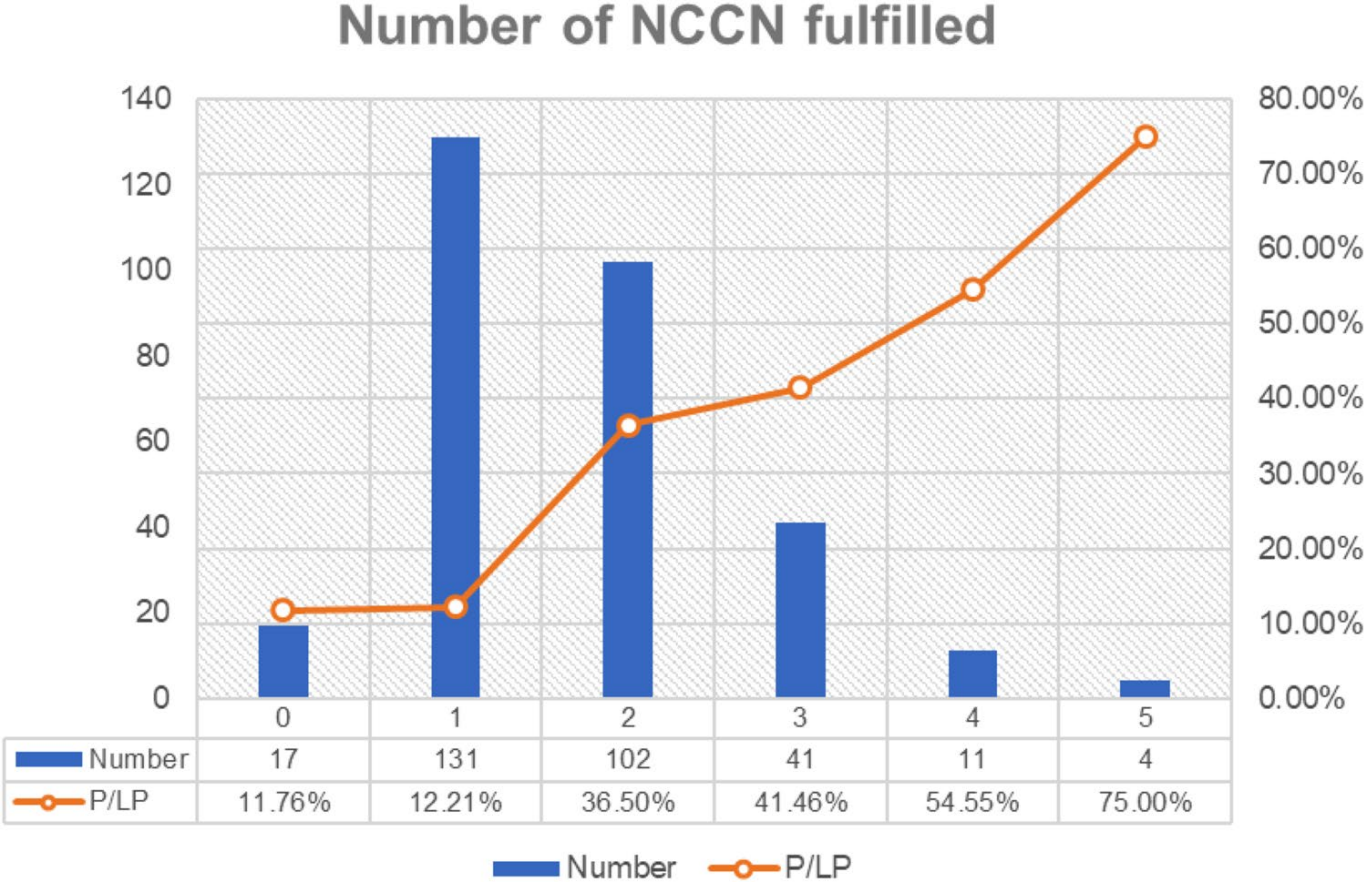
Rate of germline pathogenic/likely pathogenic variants or VUS per number of indications fulfilled from multigene panel test in Thai patients with breast cancer

Overall, patients who met at least one indication in 2019 NCCN guideline have P/LP variant detection rate varying from 27 to 40% (Fig. 2). The most frequent indication is early onset breast cancer (age of diagnosis less than 45 years). One hundred and ninety-eight patients (64.7%) fit this indication and had 27% P/LP variant detection rate. Since each patient could fulfill more than one indication in the guideline, we found that patients who matched more than one indication had higher likelihood of detecting P/LP variants. Detection rate was also increased with number of indications (Fig. 3). We found that patients matched 4 and 5 indications in 2019 NCCN guidelines had 54.6% and 75% detection rate, respectively. Interestingly, four of 18 patients (22%) who had breast cancer with second primary cancer outside hereditary breast-ovarian cancer spectrum carried germline P/LP variants. The 4 breast cancer patients had P/LP variants in *BRCA1* (with ovarian cancer and thyroid cancer), *BRCA2* (with endometrial cancer), *BRCA2* (with thyroid cancer) and *TP53* (with brain tumor).

### Multigene panel testing in patients with primary ovarian cancer, pancreatic cancer and prostate cancer

Overall, 62 patients with primary ovarian cancer had fulfilled the NCCN 2019 guideline by its specific tumor type. We further divided patients with ovarian cancer into 2 groups by the presence of breast cancer. We found that 15 of 43 patients (34.8%) in primary ovarian cancer without breast cancer harbored P/LP variants. Meanwhile, 8 of 19 patients (42.1%) with both primary ovarian cancer and breast cancer were tested positive for P/LP variants. We also found that 12 of 19 patients tested positive for P/LP variants had their tumor histopathology read as high-grade serous cystadenocarcinoma.

Patients with pancreatic cancer were also clinically indicated by NCCN 2019 guideline. Only one of 14 pancreatic cancer patients have colon cancer as another primary malignancy. Only one of 14 pancreatic cancer patients had their histopathology read as neuroendocrine tumor. Two patients with *BRCA2* P/LP variants were sporadic cases of adenocarcinoma of pancreatic cancer.

Prostate cancer patients that warranted further genetic test by NCCN 2019 guideline were described as metastatic prostate cancer or having high-grade prostate cancer (Gleason score ≥ 7) with family history of certain cancer. In our 7 patients with prostate cancer, there were 3 patients who had evidence of metastasis, and 4 patients who had Gleason score ≥ 7). Patient with *BRCA1* P/LP variants had Gleason score 6 with family history of gastric, thyroid, prostate and pancreatic cancers. The other prostate cancer patient with *BRCA2* P/LP variants had Gleason score 9, with another primary cancer including male breast cancer and squamous cell carcinoma of esophagus. He had no family history of cancer.

## Discussion

Breast cancer is one of the common cancers associated with heritable mutations. Identifying germline mutation in those patients provides great benefit on treatment selection, prophylactic and screening options for both the patients and their at-risk family members. For the first time, this study provided prevalence and landscape of germline P/LP variants among Thai patients with breast, ovarian, pancreatic and prostate cancers who were clinically indicated for genetic test by the National Comprehensive Cancer Network (NCCN) 2019 guidelines. Germline P/LP variants were detected in 24% of breast cancer and 37% of ovarian cancer patients.

The mutation frequency observed in our cancer patients was significantly higher than Western patients who underwent genetic testing with similar clinical indication. In 2018, the rate of P/LP variants from multigene-sequencing performed by laboratories across the US in higher-risk patients determined by NCCN guideline was 12.5% [7]. There are some explanations for this double in detection rate in Thai population. First, as both genetic and lifestyle factors are associated with an increased risk of breast cancer, many lifestyle factors such as hormonal use, obesity, and alcohol consumption among Asian population are less prevalent than Western counterpart. It is therefore possible that genetic factor could play more role on cancer susceptibility in Thai patients whose clinical phenotypes were not typically conformed to common sporadic cancer. Next, it had been noted that approximately half of the patients in this study fulfilled more than one NCCN 2019 indication. Our study could represent patients with higher risk profile than other studies.

Data of multigene sequencing in breast cancer in Asian population was limited, and most study did not select patients based on established guideline. One multi-centered study in China enrolled participants based on the Breast Cancer Diagnosis and Treatment Guidelines and Standards (Chinese Cancer Society, V2015), of which the criteria were comparable to the NCCN 2019. The frequency of germline P/LP variants identified in China cohort (23.8%) and our study (23.9%) were similarly high [11], as well as the proportion of *gBRCA* variants (71% vs 79.5%). Patients from Chinese cohort who fulfilled more than one indication also had higher frequency of germline P/LP variants (43.2%), in consistent with this study (40.0%).

The overall frequency of germline P/LP variants in ovarian cancer was 37%. *gBRCA* variants accounted for 57.5% of all P/LP variants. The rate was considerably higher than the US patients with ovarian cancer [12] (overall frequency of 13.4%, *gBRCA* variants accounted for 50.5% of all P/LP variants). Data of multigene sequencing in Asian ovarian cancer patients was also limited. When consider only *gBRCA* variants in an Asian population, the detection rate in Japanese ovarian cancer patients was 14.7% [13] while it was found in 22.4% of Chinese ovarian cancer patients [14]. Expansion of genetic test beyond *BRCA1/2* nearly double the rate of finding germline P/LP variants, thus multigene panel test should be encouraged in ovarian cancer. The pathological report of high-grade serous cystadenocarcinoma contributed to 63.2% of all patients with positive P/LP variants. This data supports the concept that multigene panel test should be carried out in all patients with ovarian cancer regardless of their pathological finding [13].

An observed rate of 14.3% in pancreatic cancer (*n* = 14) was also comparable with previous study in pancreatic cancer (10.5%) [12]. The P/LP variants detection rate in pancreatic cancer and prostate cancer from our study should be carefully interpreted due to the limitation in study number. Further study in these cancer types is warranted. We reported *gBRCA* as a secondary finding in 3 patients with colon cancer, 1 patient with nasopharyngeal cancer and 1 patient with lung cancer (Table 2). Previous report from the US estimated the frequency of *gBRCA* variants to be 1.6% in colon/stomach cancer and 2.6% in colon/endometrial cancer [12]. The secondary finding detection rate of genes for hereditary breast-ovarian cancer in colorectal cancer patients should be reviewed in the future when the number of testing is sufficient.

The overall rate of VUS in our cancer patients was approximately 40%. This rate was comparable to VUS rate among different ethnic groups (23.7% in white, 44.5% in African-American, and 50.9% in Asian) [7]. The prevalence of VUS in *BRCA1*/*2* in this study (7.2%) was lower than previously published report (15–21%) [15]. The decrease in VUS was likely due to an increase in functional studies in recent year, and the availability of genomic data in Asian population [16]. A study from South Korea showed that most VUS in *BRCA1*/*2* (57%) remained unchanged and only 2.7% of VUS was reclassified as likely pathogenic [17]. The reclassification of identified variants in this study remains to be seen. Some identified variants are notable for further study because they almost fulfill the ACMG 2015 guideline to be classified as pathogenic or likely pathogenic [10]. There were 8 pLOF variants (Table 3) which included start codon loss in *APC* and *RECQL*. Although there were many reports of start codon loss in other diseases [18, 19], initiation codon loss in *APC*, a well-known gene, had never been reported in colorectal cancer patients [20]. Additional genomic data and functional validation might help reclassification of VUS in the future.

Absence of copy number variations of *BRCA1* and *BRCA2* in our cohort may suggest that prevalence of large deletion/duplication in *gBRCA* among Thai patients is not as common as other population [21].

### Utilization of NCCN guideline in Thai and Asian population

The prevalence of finding germline P/LP variants in each specified indication from NCCN guideline 2019 ranged between 27% and 40% (Fig. 1). Among breast cancer patients, the rate was highest (38%) in breast cancer patient with personal or family history of primary malignancy in hereditary breast ovarian cancer spectrum. Breast cancer patients with another primary cancer not in hereditary breast ovarian cancer spectrum also had high rate of germline P/LP variants (22%; *n* = 18). This clinical scenario could be added as testing indication in Thai or Asian patients with pretest probability over 10%, the cut-off that was proposed to be cost-effective in various population [22]. The rate of detecting P/LP variants did positively correlate with number of indications fulfilled (Fig. 2). This warrants a strong recommendation of providing germline genetic test in patient with multiple indication fulfilled. Germline genetic testing guideline for breast ovarian cancer could be adopted from Western guideline/recommendation as a framework with some modification to fully maximize the clinical benefit of genetic testing, while maintaining appropriate cost-effectiveness for Asian population.

## Conclusion

We reported a high frequency of P/LP variants from multigene panel sequencing in Thai patients with breast, ovarian, pancreatic and prostate cancers that fulfilled NCCN 2019 indication for germline genetic testing. The rate of VUS and the number of identified novel variants were high and reflected the need to include more Asian or Thai dataset in genomic database. The results from this study warrant the incorporation of genetic test, particularly multigene panel test, and establishment of testing guidelines in management of breast cancer and ovarian cancer in Thai and Asian population.

## Data Availability

The data that support the findings of this study are available from the corresponding author upon reasonable request.

## Abbreviations

*BRCA1*/*2 *: *BRCA1* And *BRCA2*
*gBRCA*: Germline pathogenic variants in *BRCA1* or *BRCA2*
NCCN: National Comprehensive Cancer Network
P/LP variants: Pathogenic variants or likely pathogenic variants
VUS: Variants of undetermined significance

## References

[1] Siegel RL, Miller KD (2020). Jemal A (2020) Cancer statistics. CA Cancer J Clin.

[2] Apostolou P, Fostira F (2013). Hereditary breast cancer: the era of new susceptibility genes. Biomed Res Int.

[3] Kemp Z, Turnbull A, Yost S, Seal S, Mahamdallie S, Poyastro-Pearson E, Warren-Perry M, Eccleston A, Tan MM, Teo SH, Turner N, Strydom A, George A, Rahman N (2019). Evaluation of cancer-based criteria for use in mainstream BRCA1 and BRCA2 genetic testing in patients with breast cancer. JAMA Netw Open.

[4] Armstrong N, Ryder S, Forbes C, Ross J, Quek RG (2019). A systematic review of the international prevalence of BRCA mutation in breast cancer. Clin Epidemiol.

[5] Shiovitz S, Korde LA (2015). Genetics of breast cancer: a topic in evolution. Ann Oncol.

[6] Piffer A, Luporsi E, Mathelin C (2018). PALB2, a major susceptibility gene for breast cancer. Gynecol Obstet Fertil Senol.

[7] Kurian AW, Ward KC, Hamilton AS, Deapen DM, Abrahamse P, Bondarenko I, Li Y, Hawley ST, Morrow M, Jagsi R, Katz SJ (2018). Uptake, results, and outcomes of germline multiple-gene sequencing after diagnosis of breast cancer. JAMA Oncol.

[8] D'Andrea E, Marzuillo C, De Vito C, Di Marco M, Pitini E, Vacchio MR, Villari P (2016). Which BRCA genetic testing programs are ready for implementation in health care? A systematic review of economic evaluations. Genet Med.

[9] Euhus DM, Smith KC, Robinson L, Stucky A, Olopade OI, Cummings S, Garber JE, Chittenden A, Mills GB, Rieger P, Esserman L, Crawford B, Hughes KS, Roche CA, Ganz PA, Seldon J, Fabian CJ, Klemp J, Tomlinson G (2002). Pretest Prediction of BRCA1 or BRCA2 Mutation by Risk Counselors and the Computer Model BRCAPRO. JNCI.

[10] Richards S, Aziz N, Bale S, Bick D, Das S, Gastier-Foster J, Grody WW, Hegde M, Lyon E, Spector E, Voelkerding K, Rehm HL (2015). Standards and guidelines for the interpretation of sequence variants: a joint consensus recommendation of the American College of Medical Genetics and Genomics and the Association for Molecular Pathology. Genet Med.

[11] Li JY, Jing R, Wei H, Wang M, Xiaowei Q, Liu H, Jian L, Ou JH, Jiang WH, Tian FG, Sheng Y, Li HY, Xu H, Zhang RS, Guan AH, Liu K, Jiang HC, Ren Y, He JJ, Huang W, Liao N, Cai X, Ming J, Ling R, Xu Y, Hu CY, Zhang J, Guo B, Ouyang L, Shuai P, Liu Z, Zhong L, Zeng Z, Zhang T, Xuan Z, Tan X, Liang J, Pan Q, Chen L, Zhang F, Fan LJ, Zhang Y, Yang X, BoLi J, Chen C, Jiang J (2019). Germline mutations in 40 cancer susceptibility genes among Chinese patients with high hereditary risk breast cancer. Int J Cancer.

[12] Susswein LR, Marshall ML, Nusbaum R, Vogel Postula KJ, Weissman SM, Yackowski L, Vaccari EM, Bissonnette J, Booker JK, Cremona ML, Gibellini F, Murphy PD, Pineda-Alvarez DE, Pollevick GD, Xu Z, Richard G, Bale S, Klein RT, Hruska KS, Chung WK (2016). Pathogenic and likely pathogenic variant prevalence among the first 10,000 patients referred for next-generation cancer panel testing. Genet Med.

[13] Enomoto T, Aoki D, Hattori K, Jinushi M, Kigawa J, Takeshima N, Tsuda H, Watanabe Y, Yoshihara K, Sugiyama T (2019). The first Japanese nationwide multicenter study of BRCA mutation testing in ovarian cancer: CHARacterizing the cross-sectionaL approach to Ovarian cancer geneTic TEsting of BRCA (CHARLOTTE). Int J Gynecol Cancer.

[14] Li A, Xie R, Zhi Q, Deng Y, Wu Y, Li W, Yang L, Jiao Z, Luo J, Zi Y, Sun G, Zhang J, Shi Y, Liu J (2018). BRCA germline mutations in an unselected nationwide cohort of Chinese patients with ovarian cancer and healthy controls. Gynecol Oncol.

[15] Eccles BK, Copson E, Maishman T, Abraham JE, Eccles DM (2015). Understanding of BRCA VUS genetic results by breast cancer specialists. BMC Cancer.

[16] Findlay GM, Daza RM, Martin B, Zhang MD, Leith AP, Gasperini M, Janizek JD, Huang X, Starita LM, Shendure J (2018). Accurate classification of BRCA1 variants with saturation genome editing. Nature.

[17] So MK, Jeong TD, Lim W, Moon BI, Paik NS, Kim SC, Huh J (2019). Reinterpretation of BRCA1 and BRCA2 variants of uncertain significance in patients with hereditary breast/ovarian cancer using the ACMG/AMP 2015 guidelines. Breast Cancer.

[18] Jinda W, Poungvarin N, Taylor TD, Suzuki Y, Thongnoppakhun W, Limwongse C, Lertrit P, Suriyaphol P, Atchaneeyasakul LO (2016). A novel start codon mutation of the MERTK gene in a patient with retinitis pigmentosa. Mol Vis.

[19] Sargiannidou I, Kim GH, Kyriakoudi S, Eun BL, Kleopa KA (2015). A start codon CMT1X mutation associated with transient encephalomyelitis causes complete loss of Cx32. Neurogenetics.

[20] DeRycke MS, Gunawardena S, Balcom JR, Pickart AM, Waltman LA, French AJ, McDonnell S, Riska SM, Fogarty ZC, Larson MC, Middha S, Eckloff BW, Asmann YW, Ferber MJ, Haile RW, Gallinger S, Clendenning M, Rosty C, Win AK, Buchanan DD, Hopper JL, Newcomb PA, Le Marchand L, Goode EL, Lindor NM, Thibodeau SN (2017). Targeted sequencing of 36 known or putative colorectal cancer susceptibility genes. Mol Genet Genomic Med.

[21] Rebbeck TR, Friebel TM, Friedman E, Hamann U, Huo D, Kwong A, Olah E, Olopade OI, Solano AR, Teo SH, Thomassen M, Weitzel JN, Chan TL, Couch FJ, Goldgar DE, Kruse TA, Palmero EI, Park SK, Torres D, van Rensburg EJ, McGuffog L, Parsons MT, Leslie G, Aalfs CM, Abugattas J, Adlard J, Agata S, Aittomäki K, Andrews L, Andrulis IL, Arason A, Arnold N, Arun BK, Asseryanis E, Auerbach L, Azzollini J, Balmaña J, Barile M, Barkardottir RB, Barrowdale D, Benitez J, Berger A, Berger R, Blanco AM, Blazer KR, Blok MJ, Bonadona V, Bonanni B, Bradbury AR, Brewer C, Buecher B, Buys SS, Caldes T, Caliebe A, Caligo MA, Campbell I, Caputo SM, Chiquette J, Chung WK, Claes KBM, Collée JM, Cook J, Davidson R, de la Hoya M, De Leeneer K, de Pauw A, Delnatte C, Diez O, Ding YC, Ditsch N, Domchek SM, Dorfling CM, Velazquez C, Dworniczak B, Eason J, Easton DF, Eeles R, Ehrencrona H, Ejlertsen B, Engel C, Engert S, Evans DG, Faivre L, Feliubadaló L, Ferrer SF, Foretova L, Fowler J, Frost D, Galvão HCR, Ganz PA, Garber J, Gauthier-Villars M, Gehrig A, Gerdes AM, Gesta P, Giannini G, Giraud S, Glendon G, Godwin AK, Greene MH, Gronwald J, Gutierrez-Barrera A, Hahnen E, Hauke J, Henderson A, Hentschel J, Hogervorst FBL, Honisch E, Imyanitov EN, Isaacs C, Izatt L, Izquierdo A, Jakubowska A, James P, Janavicius R, Jensen UB, John EM, Vijai J, Kaczmarek K, Karlan BY, Kast K, Investigators K, Kim SW, Konstantopoulou I, Korach J, Laitman Y, Lasa A, Lasset C, Lázaro C, Lee A, Lee MH, Lester J, Lesueur F, Liljegren A, Lindor NM, Longy M, Loud JT, Lu KH, Lubinski J, Machackova E, Manoukian S, Mari V, Martínez-Bouzas C, Matrai Z, Mebirouk N, Meijers-Heijboer HEJ, Meindl A, Mensenkamp AR, Mickys U, Miller A, Montagna M, Moysich KB, Mulligan AM, Musinsky J, Neuhausen SL, Nevanlinna H, Ngeow J, Nguyen HP, Niederacher D, Nielsen HR, Nielsen FC, Nussbaum RL, Offit K, Öfverholm A, Ong KR, Osorio A, Papi L, Papp J, Pasini B, Pedersen IS, Peixoto A, Peruga N, Peterlongo P, Pohl E, Pradhan N, Prajzendanc K, Prieur F, Pujol P, Radice P, Ramus SJ, Rantala J, Rashid MU, Rhiem K, Robson M, Rodriguez GC, Rogers MT, Rudaitis V, Schmidt AY, Schmutzler RK, Senter L, Shah PD, Sharma P, Side LE, Simard J, Singer CF, Skytte AB, Slavin TP, Snape K, Sobol H, Southey M, Steele L, Steinemann D, Sukiennicki G, Sutter C, Szabo CI, Tan YY, Teixeira MR, Terry MB, Teulé A, Thomas A, Thull DL, Tischkowitz M, Tognazzo S, Toland AE, Topka S, Trainer AH, Tung N, van Asperen CJ, van der Hout AH, van der Kolk LE, van der Luijt RB, Van Heetvelde M, Varesco L, Varon-Mateeva R, Vega A, Villarreal-Garza C, von Wachenfeldt A, Walker L, Wang-Gohrke S, Wappenschmidt B, Weber BHF, Yannoukakos D, Yoon SY, Zanzottera C, Zidan J, Zorn KK, Hutten Selkirk CG, Hulick PJ, Chenevix-Trench G, Spurdle AB, Antoniou AC, Nathanson KL (2018). Mutational spectrum in a worldwide study of 29,700 families with BRCA1 or BRCA2 mutations. Hum Mutat.

[22] Manchanda R, Patel S, Gordeev VS, Antoniou AC, Smith S, Lee A, Hopper JL, MacInnis RJ, Turnbull C, Ramus SJ, Gayther SA, Pharoah PDP, Menon U, Jacobs I, Legood R (2018). Cost-effectiveness of population-based BRCA1, BRCA2, RAD51C, RAD51D, BRIP1, PALB2 mutation testing in unselected general population women. J Natl Cancer Inst.

